# Acute caffeine ingestion reduces insulin sensitivity in healthy subjects: a systematic review and meta-analysis

**DOI:** 10.1186/s12937-016-0220-7

**Published:** 2016-12-28

**Authors:** Xiuqin Shi, Wenhua Xue, Shuhong Liang, Jie Zhao, Xiaojian Zhang

**Affiliations:** Department of Pharmacy, The First Affiliated Hospital of Zhengzhou University, No. 43 Daxue Road, 450052 Zhengzhou, People’s Republic of China

**Keywords:** Caffeine, Insulin sensitivity, Diabetes, Risk factor

## Abstract

**Background:**

According to previous meta-analyses, coffee consumption reduces the risk of type 2 diabetes mellitus. However, the underlying mechanism remains unknown. Whether caffeine, the key ingredient in coffee, has a beneficial effect on the glycemic homeostasis and the anti-diabetic effect is particularly controversial. The aim of this study was to summarize the effect of acute caffeine ingestion on insulin sensitivity in healthy men.

**Methods:**

A comprehensive literature search for papers published before April 2016 was conducted in EMBASE, PubMed, and Cochrane Library databases. Randomized controlled trials (RCTs) that investigated the effect of caffeine on insulin sensitivity in healthy humans without diabetes were included. A random effects meta-analysis was conducted using Review Manager 5.3.

**Results:**

The search yielded 7 RCTs in which caffeine intake was the single variant. Compared with placebo, caffeine intake significantly decreased the insulin sensitivity index, with a standardized mean difference of −2.06 (95% confidence interval −2.67 to −1.44, I^2^ = 49%, *P* for heterogeneity = 0.06).

**Conclusion:**

Acute caffeine ingestion reduces insulin sensitivity in healthy subjects. Thus, in the short term, caffeine might shift glycemic homeostasis toward hyperglycemia. Long-term trials investigating the role of caffeine in the anti-diabetic effect of coffee are needed.

## Introduction

The worldwide prevalence of type II diabetes has doubled in the previous two decades; this, in combination with the high disability and mortality rates has contributed to the serious effects of type II diabetes on human health [1]. A systematic analysis of health examination surveys and epidemiological studies estimated that the overall age-standardized incidence of diabetes was approximately 9.2% for women and 9.8% for men [2]. Prevention of diabetes through the adoption of healthy dietary habits is particularly important for the effective management of hyperglycemia [3, 4].

Coffee is one of the most widely consumed beverages globally. Different amounts of coffee are habitually consumed to different countries or by people of different races; residents of the Netherlands and Finland are among the largest coffee drinkers, and Caucasians tend to consume more coffee than other races. Evidence is accumulating that habitual coffee consumption is linked to a reduced incidence of type II diabetes [5], and epidemiological studies conducted with people of different races have shown that coffee consumption is inversely correlated with the incidence of type II diabetes [6–16]. Importantly, higher consumption of all coffee products, caffeinated coffee, and decaffeinated coffee was associated with a lower risk of total mortality [17, 18].

Various mechanisms have been proposed regarding the protective effect of long-term coffee consumption against type II diabetes. Kemfp et al. reported that coffee consumption might have beneficial effects on subclinical inflammation and high-density lipoprotein cholesterol [19]. Kagami et al. showed that caffeine protects pancreatic beta cells against streptozocin toxicity [20]. Nevertheless, whether caffeine alone influences glycemic control, which is closely linked with diabetes-related micro- or macro-vascular complications [21], remains unknown; this knowledge would provide insight into the role of caffeine in the putative relationship between coffee consumption and the risk of diabetes.

Caffeine, a mildly psycho-active chemical, is one of the most commonly consumed “drugs”, with an average daily individual intake of approximately 300 mg from dietary sources such as coffee, tea, soft drinks, chocolate, and energy drinks, among which coffee is the predominant source. Caffeine is one of the main components in coffee and is also used as an additive in more than 60% of available soft drinks [22]. Tea and other beverages that contain caffeine also reportedly protect against type II diabetes [23]. However, the underlying mechanism remains unclear, and the results of bedside and bench-based research are controversial [19, 24, 25]. Because type II diabetes is characterized by impaired insulin sensitivity, caffeine might play an important role in the protective effect of coffee against type II diabetes.

The current meta-analysis aimed to investigate the effect of caffeine alone on insulin sensitivity in healthy subjects. Whitehead et al. conducted a systematic review of randomized controlled trials (RCTs) of the effects of caffeine on glycemic control in people with diabetes mellitus [26]. However, the implications of the results might be limited, because the beneficial effect of coffee consumption for diabetes is supposedly more preventive than healing. Hence, data from healthy subjects are needed to investigate the protective effect of coffee against diabetes. Ding et al. conducted a systematic review of the association between caffeinated/decaffeinated coffee consumption and the risk of type II diabetes [18]. Similarly, Jiang et al. conducted a meta-analysis of prospective studies of the relationship between coffee/caffeine intake and the incidence of type II diabetes [17]. There is some discrepancy between these reviews regarding the effect of caffeine on diabetes prevalence. Because very few systematic reviews and meta-analyses have been conducted for RCTs on the association between caffeine alone and glycemic control in healthy subjects, the current meta-analysis could be significant for clarifying the inverse association between coffee consumption and type II diabetes.

## Methods

### Search strategy

The PubMed (1960 to April 2016; http://www.ncbi.nlm.nih.gov/pubmed/), EMBASE (1980 to April 2016; http://www.embase.com/), and Cochrane Library (1985 to April 2016; http://www.cochrane.org/) databases were searched for RCTs that examined the effects of caffeine on insulin sensitivity in healthy humans. The following key words in the title/abstract were used: *caffeine* and *glucose, glycemic control, insulin, or insulin sensitivity*. The search was restricted to human clinical trials. In addition, the reference lists from selected reports were reviewed for further relevant studies. The systematic review was planned, conducted, and reported according to the PRISMA statement [27]. The review protocol was registered in the PROSPERO International Prospective Register of Systematic Reviews (CRD42016041224).

### Study selection

The following criteria were used for study selection: 1) subjects consumed a caffeine-containing beverage before ≥2-week washout during which no caffeine-containing products were consumed; 2) the study was an RCT with a parallel or crossover design conducted with healthy humans; 3) insulin sensitivity was calculated using standard deviations; 4) the only variant between the experimental and control groups was caffeine administration. The quality of the selected studies, regarding the risk of bias, was assessed using the computer program Review Manager (RevMan), Version 5.3 (The Nordic Cochrane Centre, The Cochrane Collaboration, Copenhagen).

**Fig. 1:**
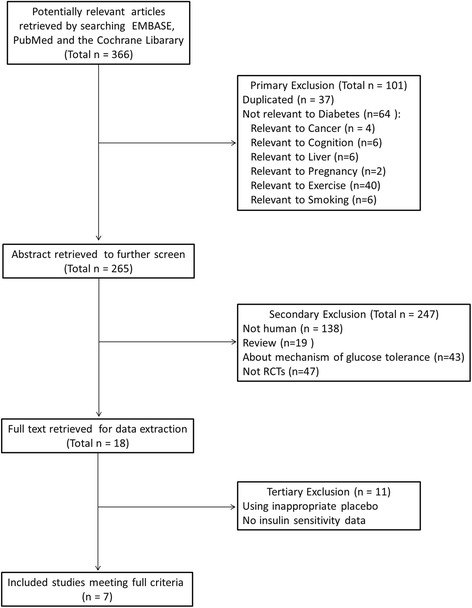
Selection process of the identified articles

Of the 366 studies identified in the initial search, the first review excluded 101 publications for not being relevant or a duplicate study, the second review excluded 247 publications because they did not describe human clinical trials, and the third review excluded 11 publications that did not include appropriate insulin sensitivity data. Therefore, 7 studies involving 70 participants were included (Fig. 1). The selected placebo was reviewed to assure that caffeine intake was the only variant. One study by Greenberg et al. [28] used two pairs of treatments: caffeine vs. placebo and caffeinated coffee vs. decaffeinated coffee. Only data from the latter pair were included because coffee consumption occurs more frequently than administration of pure caffeine in daily life.

### Data extraction

Study features, including authors & publication year, sample size (male: female), study design, and other relevant information were collected, in addition to the insulin sensitivity index (Table 1).

**Table 1:** Characteristics of the included studies for the meta-analysis of insulin sensitivity

Author Year	Study design	Subjects (n/m:f)	Subject characteristics (Age, body mass index [kg/m^2^], other information)	Duration of caffeine abstention	Caffeine dose Mode of administration Placebo	Insulin sensitivity assessment method
Battram 2006	Randomized Double-blind Crossover	11 11:0	23.3 ± 0.6 y Healthy, non-smokers Body weight: 76.4 ± 1.9 kg Percent body fatness 15.6 ± 2.3	7 days	4.45 mg/kg caffeine Orally Dextrose	OGTT ^a^Matsuda & DeFronzo Equation [39]
Greenberg 2010	Randomized Single-blind Crossover	11 11:0	23.5 ± 5.7 y 23.6 ± 4.2 kg/m^2^ 1 week prior: maintain stable diet, exercise, and alcohol intake No caffeinated drinks, foods, or medications No smoking No alcohol or exercise 48 h prior	48 h	6 mg/kg caffeine Orally Caffeine *vs* warm water Caffeinated coffee *vs* decaffeinated coffee	OGTT Matsuda & DeFronzo Equation [39]
Keijzers 2002	Randomized Double-blind Crossover	12 6:6	20–28 y Nonsmoking 21.9 ± 2.7 kg/m^2^ (lean)	72 h	3 mg/kg caffeine Intravenously NaCl 0.9%	Hyperinsulinemic- euglycemic glucose clamp
Moisey 2008	Randomized Single-blind Crossover	10 10:0	23.3 ± 1.1 y 25.3 ± 1.05 kg/m^2^ Non-smokers, healthy	48 h	5 mg/kg caffeinated coffe Orally Decaffeinated coffee	OGTT Belfiore formula [39]
Moisey 2010	Randomized Double-blind Crossover	10 10:0	23.3 ± 1 y 23.4 ± 0.8 kg/m^2^ Non-smokers, healthy 3/10 Non-caffeine users	48 h	5 mg/kg caffeinated coffee Orally Decaffeinated coffee	OGTT Matsuda & DeFronzo Equation [39]
Petrie 2004	Randomized Double-blind Crossover	9 9:0	19–34 y No history of disease (diabetes mellitus) No drug or alcohol abuse Sedentary 34.0 ± 1.0 kg/m^2^ (obese)	48 h	5 mg/kg caffeine Orally Dextrose	OGTT Matsuda & DeFronzo Equation [39]
Thong 2002	Randomized Double-blind Crossover	7 7:0	24 ± 1 y 23 cpe ± 1 kg/m^2^ Lean	48 h	5 mg/kg caffeine Orally 5 mg/kg dextrose	OGTT Matsuda & DeFronzo Equation [39]

^a^Matsuda and DeFronzo equation $ISI=\frac{10000}{\surd FPG\times FPI\Big)\times \left(\overline{G}\times \overline{I}\right)}$ where FPG is fasting plasma glucose, FPI is fasting plasma insulin, $\overline{G}$ is mean plasma glucose concentration during OGTT, and *Ī* is mean plasma insulin concentration during OGTT

### Statistical analysis

The current meta-analysis was performed using the computer program RevMan 5.3. Copenhagen. Heterogeneity between trial results was tested using a standard *χ* ^2^ test. The I^2^ parameter was used to quantify any inconsistency, and I^2^ > 50% was considered to represent substantial heterogeneity [29]. Because of the different methodologies adopted by individual researchers, a random-effects model was used to assess the standardized mean differences (SMD) and 95% confidence intervals (CIs) in insulin sensitivity. Forest plots were generated to illustrate the study-specific effect sizes and a 95% CIs. Data extraction was independently conducted by the two co-first authors, with disagreements resolved by consensus.

## Results

### Study characteristics

The trial characteristics are shown in Table 1. The samples varied in size from 7 to 12 subjects. All the studies were of a single- or double-blind, randomized crossover design. Six of the seven trials [28, 30–34] included male subjects, and the sample in the remaining trial [35] was 50% men. All of the subjects had no history of diabetes or other metabolic diseases. All of the publications, except those by Petrie et al. [31] and Thong et al. [30], mentioned that the subjects were non-smokers. The subjects in 6 studies [28, 30, 32–35] were lean (BMI ≤ 25 kg/m^2^). The remaining trial conducted by Petrie et al. [31] examined the effects of caffeine ingestion and nutrition/exercise intervention in obese subjects (BMI 34.0 ± 1.0 kg/m^2^). All of the trials used a washout time of ≥ 48 h. The most commonly used caffeine dose was 5 mg caffeine/kg body weight [30, 31, 33, 34], while Battram et al. [32] used 4.45 mg/kg, Greenberg et al. [28] used 6 mg/kg and Keijzer used 3 mg/kg.

### Data quality and risk of bias

**Fig. 2:**
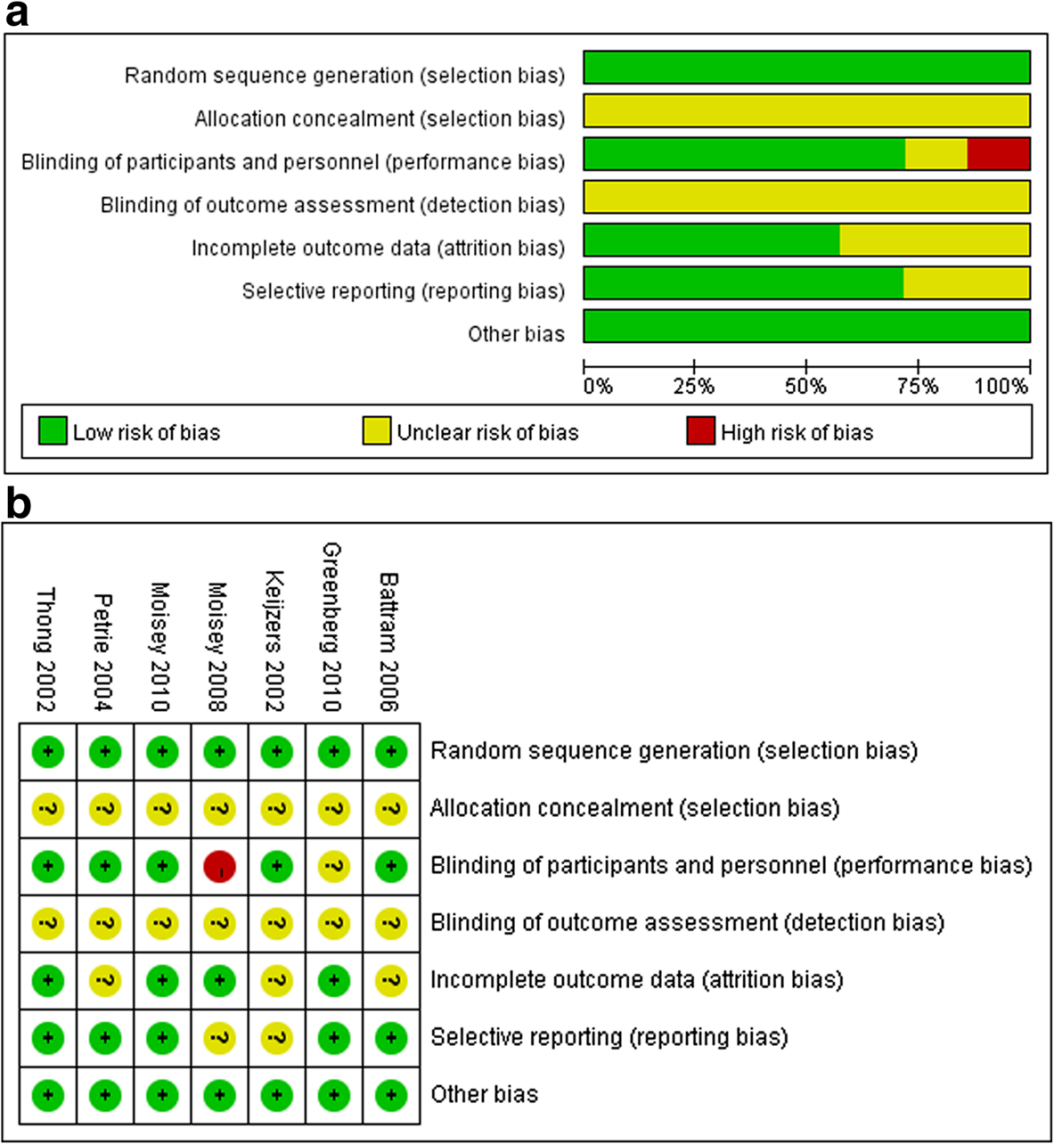
Risk of bias assessment tool: (**a**) risk of bias graph; (**b**) risk of bias summary. Across trials, information is either from trials at a low risk of bias (*green*), at an unclear risk of bias (*yellow*), or at a high risk of bias (*red*)

All of the included studies used a randomized, crossover design, reducing selection bias. However, the studies did not describe allocation concealment. In the study by Greenberg et al., the researchers were blinded to the participants’ group. Because the researchers assigning the tested beverages were different from those who collected and analyzed the outcome data, the blinding of participants and personnel was rated unclear in relation to performance bias (Fig. 2a and b). In contrast, the principle researchers were blinded to all of the experimental treatments, but the subjects were not blinded to the cereal treatment in the study conducted by Moisey et al. [33]. Therefore, the knowledge that they were consuming cereal with a low glycemic index could have resulted in a substantial placebo effect; as a result, the risk of performance bias was rated as high. There were no specific statements about the blinding of the outcome assessment, resulting in unclear risk of detection bias. The measured outcomes in each included study were determined in the trial planning stage, and all but 2 reported all the pre-designed measurements, resulting in no reporting bias. No other bias was detected in the 7 studies.

### Caffeine consumption and insulin sensitivity in healthy subjects

**Fig. 3:**
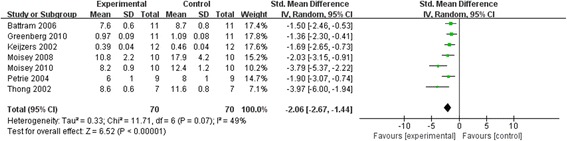
Forest plot showing the pooled standardized mean difference (SMD) and 95% confidence interval (CI) for insulin sensitivity for 7 randomized controlled trials. For each study of caffeine, the shaded square represents the point estimate of the intervention effect. The horizontal line joins the lower and upper limits of the 95% CI of these effects. The area of the shaded square reflects the relative weight of the study in the respective meta-analysis. The diamond at the bottom of the graph represents the pooled SMD with the 95% CI for the seven trials. Experimental: caffeine group; Control: control group; I^2^: Inconsistency

Figure 3 demonstrates the effect of caffeine consumption on insulin sensitivity in healthy subjects, as determined in the meta-analysis [28, 30–36]. The included studies adopted two different strategies to determine the insulin sensitivity index: a hyperinsulinemic-euglycemic glucose clamp and/or the Matsuda and DeFronzo equation [37] after an oral glucose tolerance test (OGTT). Keijzers et al. [35] was the first to use a hyperinsulinemic-euglycemic glucose clamp to calculate the insulin sensitivity in 12 non-smoking, lean, normotensive, healthy volunteers. Owing to the different methodologies, we used the SMD to measure the effect, inverse variance as the statistical method, and random effects as the analytic model.

All trials consistently reported a reduction in insulin sensitivity with caffeine consumption. Compared with placebo, caffeine intake significantly decreased the insulin sensitivity index (SMD −2.06, 95% CI −2.67 to −1.44, I^2^ = 49%, *P* for heterogeneity = 0.07). The trial performed by Petrie et al. [31] generated two sets of data for the effect of caffeine ingestion: before and after a nutrition/exercise intervention. The intervention significantly decreased body weight and percentage body fat. Subsequently, the insulin sensitivity index markedly increased from 4 ± 1 (caffeine) and 5 ± 1 (placebo) to 6 ± 1 (caffeine) and 8 ± 1 (placebo). We included the data after the nutrition and exercise intervention because the heterogeneity was lower, as compared with the other included studies.

The epidemiological evidence for an inverse association between coffee and type II diabetes is strong, while evidence is accumulating that acute caffeine intake might impair glucose metabolism. Therefore, the effect of decaffeinated coffee on glycemic control was tested by many scientists. Greenberg et al. [28] reported acutely impaired glucose metabolism by decaffeinated coffee in healthy young men. However, Battram et al. [32] found an acute enhancement of glucose metabolism by ground decaffeinated coffee, while Johnston et al. [38], Tom [39], and Van Dijk et al. [40] found no acute effect on glucose metabolism by instant decaffeinated coffee.

### Elevated glucose and insulin response after caffeine intake

A 2-h OGTT is commonly used by researchers to investigate glucose homeostasis. During the screening of the literature, we found another set of 7 studies [31–33, 36, 38, 41, 42] that explore the effect of caffeine on glucose modulation, reporting the area under the curve (AUC) for glucose and insulin. Pizzol et al. [42] tested the effects of 200 mg oral caffeine on glucose tolerance; during a 4-h OGTT, the glycemic curve was normal in both the caffeine and placebo groups until the second hour, but shifted towards the right in the caffeine group in the 2nd, 3rd, and 4th hours, compared with placebo. The AUC was separated into two parts: initial phase (≤2nd hour) and terminal phase (3rd and 4th hours). Unfortunately, the heterogeneity of these studies was too large to effectively perform a meta-analysis (I^2^ = 87%, *P* < 0.001); however, all of the studies consistently reported elevated AUCs for both glucose and insulin.

## Discussion

In the current meta-analysis, acute administration of caffeine reduced insulin sensitivity. Therefore, the inverse association between coffee and diabetes was not attributed to enhanced glucose control.

The global difference in caffeine intake could stem from multiple reasons, including different dietary habits. Residents of Western countries tend to drink more coffee, while residents of Eastern countries tend to drink more tea. Genetic reasons could also contribute; in a recent publication in *Scientific Reports*, a team of researchers reported that participants in Italy and the Netherlands with the genetic variant PDSS2 tended to drink one fewer cup of coffee per day than those without the variant [43]. Further analysis revealed PDSS2 gene expression appears to inhibit the body’s ability to break down caffeine [43]. Therefore, people with this variant would require less coffee for the same caffeine effect because the caffeine would be available in their system for a longer time.

Caffeine (1, 3, 7-trimethylxanthine) is a derivative of methylxanthine, a potentially antagonizing adenosine receptor. Data from animal models or in-vitro studies indicate that methylxanthines are involved in insulin-mediated glucose metabolism in adipose and muscle tissue [44, 45], implying a close link between caffeine and glycemic control. However, caffeine might also regulate glucose metabolism by reducing the intracellular energy status in an insulin-independent manner [46].

The current meta-analysis could be of some significance for clarifying the relationship between caffeine consumption and diabetes. In a systematic review, Ding et al. compared the trends of the association between caffeinated/decaffeinated coffee and the risk of type II diabetes. The authors reported a protective effect of decaffeinated coffee against diabetes, indicating that components of coffee other than caffeine are responsible for the putative beneficial effect. However, without data from those who consume pure caffeine, the aforementioned conclusion could be without basis. In contrast, in the meta-analysis by Jiang et al., which explored the association between coffee/caffeine intake and type II diabetes incidence, the dose-response data suggested that incremental caffeine intake had a stronger beneficial effect for type II diabetes than coffee intake, and caffeine intake provided greater protection than decaffeinated coffee intake. The results implied an indispensable role of caffeine in the protective effect against diabetes. The current meta-analysis demonstrated that acute caffeine ingestion was associated with reduced insulin sensitivity, implying that the long-term benefit of caffeine intake could be attributed to better management of calorie intake in coffee drinkers.

This review has some limitations. Restricted by the selection strategy, the selected trials all had a relatively small sample size. Nevertheless, the current results are supported by plenty of indirect evidence. Thong et al. demonstrated that caffeine induces insulin-stimulated glucose uptake in human skeletal muscle [47]. In addition, the antagonistic effects of caffeine on insulin in vivo are reportedly mediated by elevated epinephrine levels [30]. Furthermore, chronic caffeine intake reverses aging-induced insulin resistance in rats [48], highlighting the beneficial effect of long-term caffeine intake. Hence, further long-term RCTs of caffeine intake are needed to elucidate the underlying mechanism of the inverse association between coffee consumption and type II diabetes.

The significance of caffeine in diabetes is noticeable in other areas. For patients with diabetes, it is vital to be aware of decreasing blood glucose levels, because a lack of awareness about hypoglycemia is dangerous. The study by Debrah et al. found that caffeine induces a sustained decrease in middle cerebral artery velocity and augments sympathoadrenal and symptomatic responses during moderate hypoglycemia, representing a potentially useful treatment for patients with diabetes that have difficulty recognizing the onset of hypoglycemia [49].

## Conclusions

The current meta-analysis demonstrated that acute administration of caffeine attenuates insulin sensitivity in healthy subjects. It is imperative that additional evidence about the chronic effect of caffeine be collected to better understand the role of coffee in the development and prevention of type 2 diabetes.
